# Characterisation of Osteopontin in an In Vitro Model of Embryo Implantation

**DOI:** 10.3390/cells8050432

**Published:** 2019-05-09

**Authors:** Stéphane C Berneau, Peter T Ruane, Daniel R Brison, Susan J Kimber, Melissa Westwood, John D Aplin

**Affiliations:** 1 Maternal and Fetal Health Centre and Division of Developmental Biology and Medicine, Faculty of Biology, Medicine and Health, University of Manchester, Manchester Academic Health Sciences Centre, St Mary’s Hospital, Manchester M13 9WL, UK; s.berneau@mmu.ac.uk (S.C.B.); daniel.brison@manchester.ac.uk (D.R.B.); melissa.westwood@manchester.ac.uk (M.W.); john.aplin@manchester.ac.uk (J.D.A.); 2 Department of Reproductive Medicine, Old St Mary’s Hospital, Central Manchester University Hospitals NHS Foundation Trust, Manchester Academic Health Science Centre, Oxford Road, Manchester M13 9WL, UK; 3 Division of Cell Matrix Biology and Regenerative Medicine, School of Biological Sciences, Faculty of Biology Medicine and Health, University of Manchester, Michael Smith Building, Manchester M13 9PT, UK; sue.kimber@manchester.ac.uk

**Keywords:** Osteopontin, embryo implantation, endometrium

## Abstract

At the onset of pregnancy, embryo implantation is initiated by interactions between the endometrial epithelium and the outer trophectoderm cells of the blastocyst. Osteopontin (OPN) is expressed in the endometrium and is implicated in attachment and signalling roles at the embryo–epithelium interface. We have characterised OPN in the human endometrial epithelial Ishikawa cell line using three different monoclonal antibodies, revealing at least nine distinct molecular weight forms and a novel secretory pathway localisation in the apical domain induced by cell organisation into a confluent epithelial layer. Mouse blastocysts co-cultured with Ishikawa cell layers served to model embryo apposition, attachment and initial invasion at implantation. Exogenous OPN attenuated initial, weak embryo attachment to Ishikawa cells but did not affect the attainment of stable attachment. Notably, exogenous OPN inhibited embryonic invasion of the underlying cell layer, and this corresponded with altered expression of transcription factors associated with differentiation from trophectoderm (*Gata2*) to invasive trophoblast giant cells (*Hand1*). These data demonstrate the complexity of endometrial OPN forms and suggest that OPN regulates embryonic invasion at implantation by signalling to the trophectoderm.

## 1. Introduction

Pregnancy is established following embryo implantation into the endometrium, and failure at this stage occurs in the majority of natural and assisted conceptions [1,2]. Endometrial receptivity to implantation is regulated by endocrine and paracrine action, leading to differentiation of the epithelium and underlying stromal tissue to support embryo development [3,4]. Understanding the processes underpinning receptivity is therefore necessary to improve fertility treatments and to further basic understanding of early development [5]. 

The expression of osteopontin (OPN) is highly upregulated in the receptive endometrium of all placental mammals examined [6,7,8,9,10,11]. In the human endometrium, OPN is associated with epithelial, immune and vascular cells [12], and is strongly upregulated by progesterone in the mid secretory, receptive phase of the menstrual cycle [13,14]. OPN is a secreted protein with a range of functions in inflammation and extracellular matrix biology, complemented by a repertoire of variants including alternatively spliced forms, transglutaminase cross-linked oligomers, proteolytic cleavage products, glyco-, and phospho-forms [15,16]. OPN binds to cell surface receptors, including integrins and CD44, to anchor cells to the extracellular matrix. Signalling through these adhesion proteins, and co-receptors including IGF1R, EGFR and FGFR, regulates intracellular pathways controlling cell behaviour and growth [17,18]. 

The cellular architecture of the materno–foetal interface varies widely amongst mammals. In species with epitheliochorial placentation, such as sheep, extraembryonic trophoblast cells and the endometrial epithelium remain stably adherent throughout pregnancy [19]. Extensive studies in ruminants have determined that OPN dimers or multimers act as bridging ligands in integrin adhesion complexes between the endometrium and the trophoblast [20,21,22]. In species including mouse and human, implantation is invasive with trophoblast penetrating the epithelium and migrating into the differentiated stromal decidua to form the haemochorial placenta [19]. OPN secreted by the endometrium has been implicated in the attachment of the first extraembryonic lineage, the trophectoderm of the blastocyst-stage embryo, to endometrial epithelial cells during mouse implantation [23,24]. OPN also signals through focal adhesion kinase (FAK) and phosphoinositide 3-kinase (PI3K) to regulate integrin activity in the trophectoderm [25]. In addition, mouse blastocysts express their own OPN in response to oestrogen at implantation [25,26]. OPN gene knock-out does not render mice of either sex infertile [27], but there is great variation in implantation type between species, leaving open the possibility that OPN may contribute to reproductive efficiency. In particular, the interplay between its attachment and signalling roles requires delineating in order to understand OPN in invasive implantation. In humans, if extracellular OPN has a role to play, it could find an application in treating implantation failure, which occurs frequently after IVF/embryo transfer [28]. 

Studying human implantation requires in vitro models, and culturing human blastocysts with primary human endometrial epithelial cells revealed trophectoderm attachment through intercellular adhesion complexes [29]. Modelling implantation with mouse blastocysts and the human endometrial epithelial Ishikawa cell line, we revealed that Ishikawa cells signal to trophectoderm transcription factor networks to promote breaching of epithelial cells [30]. We also demonstrated upregulation of OPN and its receptor, integrin αvβ3, in Ishikawa cells surrounding the attached human blastocyst, while knock-down of either partner destabilised, but did not abolish, attachment of mouse blastocysts [24]. Further implicating OPN, we found that blocking an alternative OPN receptor, CD44, with antibodies delays attachment [31]. CD44 is also a receptor for hyaluronic acid (HA), which has been used in fertility treatments as a supplement during embryo transfer to promote implantation [32]. Here, we characterise OPN forms in Ishikawa cells and explore the effects of supplementing our model of early implantation with exogenous OPN.

## 2. Materials and Methods

### 2.1. Cell Culture

Ishikawa cells (ECACC 99040201) were cultured in Dulbecco’s Modified Eagle Medium (DMEM, Sigma, Gillingham, UK) containing 10% foetal bovine serum (Sigma), 2 mM L-glutamine, 100 µg/mL streptomycin and 100 IU/mL penicillin (Sigma) at 37 °C, 5% CO_2_.

### 2.2. Immunoprecipitation

Ishikawa cells cultured in 10 cm dishes to confluence were washed and scraped into lysis buffer (25 mM Tris pH 7.4, 150 mM NaCl, 5 mM EDTA, 1% Nonidet P-40, 0.25% sodium deoxycholate, protease inhibitor cocktail (Calbiochem, Watford, UK), and complete phosphatase inhibitor cocktail (Sigma)) before clarification by centrifugation. Lysates were incubated with 5 µg antibody (Table 1) per 0.5 mL on a rotator for 2 h at 4 °C. Washed protein G-conjugated agarose beads (Pierce) were then added to lysates followed by rotating incubation for 1 h at 4 °C. Beads were washed in lysis buffer before eluting into SDS PAGE sample buffer.

### 2.3. SDS PAGE and Western Blotting

Ishikawa cell lysates and immunoprecipitations were separated by 10% acrylamide SDS PAGE and transferred onto nitrocellulose membranes (GE Healthcare, Little Chalfont, UK). Membranes were blocked for 30 min with agitation in PBS-4% bovine serum albumin (BSA, Sigma). Incubation with primary antibody (Table 1) for 2 h in PBS-4% BSA, 0.5% tween-20 was followed by incubation with secondary antibodies (IRDye, LI-COR Biosciences) for 1 h in PBS-4% BSA, 0.5% tween-20. Membranes were washed in PBS-0.5% tween-20 after each antibody incubation and analysed using the LI-COR Odyssey infrared imaging system (LI-COR Biosciences, Cambridge, UK).

### 2.4. Mouse Embryo Collection

Mouse embryo collection was performed under UK Home Office project license PPL 70/07838, authorised by the Animal Welfare and Ethical Review Board of the University of Manchester, according to the Animal Act, 1986. Eight-week-old CD1 female mice (Charles River) were subject to intraperitoneal injection of 5 IU pregnant mare serum gonadotrophin (Intervet, Milton Keynes, UK), followed by 5 IU human chorionic gonadotrophin (Intervet) 46 h later, to induce superovulation. Mice were then housed overnight with <9-month-old CD1 male mice (Charles River). Two-cell embryos were flushed with M2 medium (Millipore, Watford, UK) from dissected oviducts 44 h later at embryonic day (E)1.5 and cultured in KSOM medium (Millipore) containing 0.4% BSA under oil (Vitrolife, Warwick, UK) at 37 °C, 5% CO_2_. At E4.5 blastocyst stage, embryos were hatched from the zona pellucida using acid Tyrode’s solution (pH 2.5, Sigma).

### 2.5. In Vitro Implantation Assay

Ishikawa cells were seeded on 2% Matrigel (Sigma)-coated glass coverslips in 24-well plates and cultured to confluency. Cells were incubated in serum-free medium (DMEM, 2 mM l-glutamine, 100 µg/mL streptomycin and 100 IU/mL penicillin) 24 h prior to co-culture with three hatched E4.5 blastocysts per well and attachment stability was assessed using an inverted phase contrast microscope (Evos XL Core, ThermoFisher, Loughborough, UK) over 48 h co-culture, as previously described [30]. To test the effects of OPN on attachment, 1 µg/mL recombinant human OPN (rhOPN, R and D Systems, Abingdon, UK) was added at the onset of co-culture (E4.5), or after 24 h co-culture (E5.5) just prior to detachment of any weakly attached embryos by gently flushing wells with medium. Co-cultures were fixed after 48 h with PBS-4% paraformaldehyde (PFA) for 20 min at room temperature and stored under PBS at 4 °C.

### 2.6. Immunofluorescence Staining

Ishikawa cells on coverslips with or without attached mouse embryos were fixed with PBS-4% PFA for 20 min, quenched with PBS-50 mM ammonium chloride for 5 min and permeabilised in PBS-0.5% Triton-X100 PBS for 6 min. These samples were incubated with primary antibodies (Table 1) in PBS at room temperature for 2 h, before washing and room temperature incubation for 1 h with PBS containing Alexa 488/568-conjugated secondary antibodies (Life Technologies, Inchinnan, UK), Alexa 568-conjugated phalloidin (Life Technologies) and DAPI (Sigma). Coverslips with Ishikawa cells only were mounted upside down on a microscope slide in a drop of Mowiol 4–88 mounting medium (Sigma) containing 3% 1,4-diazabicyclo[2.2.2]octane (Sigma). Coverslips with embryos attached to Ishikawa cells were mounted in a chamber of 3% DABCO in PBS to maintain the 3D structure of the attachment sites.

### 2.7. Fluorescence Microscopy

Fluorescence microscopy images were captured with an inverted microscope (Zeiss Axiophot, Cambridge, UK), Zen 2.0 software and the Apotome 2 module, and analysed with Zen 2.0 and ImageJ. Optical sections of cells were obtained at 0.24 µm increments, and those of embryos attached to Ishikawa cells were obtained at 2 µm increments.

### 2.8. Blastocyst RNA Extraction and Quantitative PCR

RNA was extracted from ten blastocysts per experiment using the RNeasy Micro Kit (Qiagen, Manchester, UK). Sensiscript RT kit (Qiagen) together with random 9mer primers (Agilent, Wokingham, UK) was used to perform reverse transcription (RT) reactions with 12 ng RNA. Quantitative PCRs (qPCRs) were carried out using the RT reactions along with 0.25 μM primers (Table 2) and QuantiTect SYBR green PCR kit (Qiagen). A Stratagene Mx3000p machine was used to run qPCRs with thermocycle parameters according to QuantiTect instructions (35 cycles using 58 °C annealing temperature for all primers). Stratgene MxPro analysis yielded cycle threshold (Ct) values which were used to establish expression relative to housekeeping genes (Table 2). Sample RNA- and reverse transcriptase-negative RT reactions were used as controls in qPCR reactions with all primer pairs. Dissociation curves were obtained in all qPCRs to demonstrate specific amplification.

### 2.9. Statistical Analysis

Embryo attachment data are represented as the mean ± the standard error of the mean. Independent t-test, two-way ANOVA followed by Bonferroni’s multiple comparisons post-hoc test statistical tests were performed using Prism (GraphPad, San Diego, CA, USA). 

## 3. Results

### 3.1. Biochemical Characterisation of OPN from Ishikawa Endometrial Epithelial Cells using Three Different Antibodies

**Figure 1:**
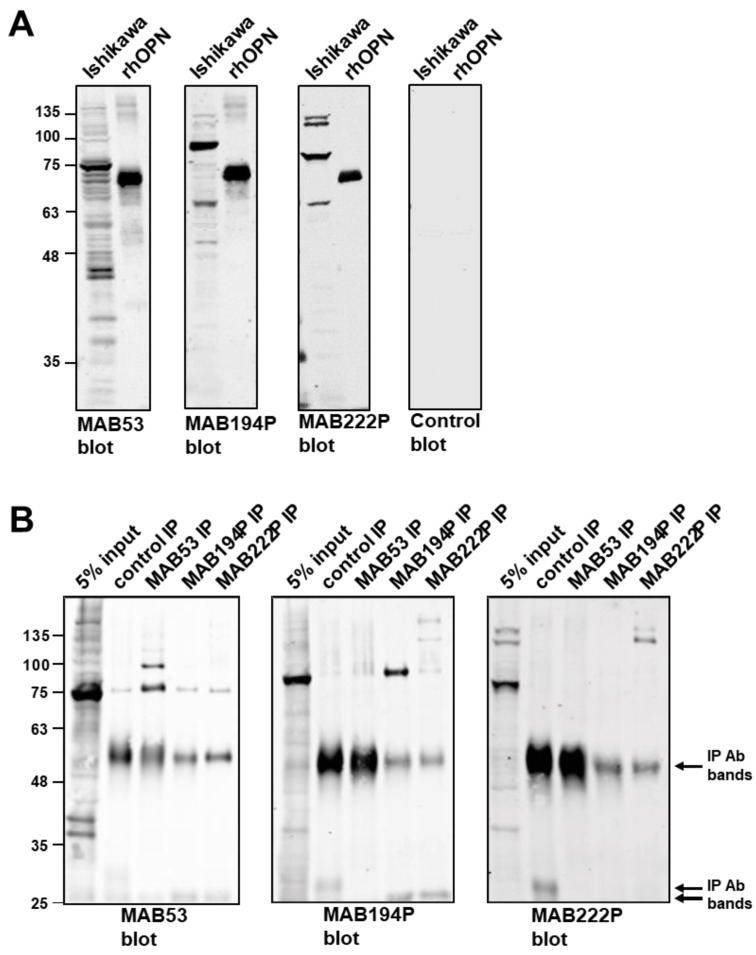
(**A**) Western blots of Ishikawa cell lysate and recombinant human OPN (rhOPN), using monoclonal antibodies MAB53, MAB194P, MAB222P and a non-specific mouse IgG control antibody. (**B**) Immunoprecipitates were produced from Ishikawa cell lysates using MAB53, MAB194P, MAB222P or control antibody before western blotting with MAB53, MAB194P and MAB222P.

Ishikawa cell lysates were probed by western blotting using three different monoclonal antibodies (MAB53, MAB194P and MAB222P). These antibodies recognised dominant bands at ~70 kDa, ~80 kDa and ~75 kDa, respectively, indicating that the corresponding epitopes are associated with distinct OPN forms (Figure 1A). Each antibody detected rhOPN commercially produced in mouse myeloma cells at ~60 kDa (Figure 1A).

Western blotting combined with immunoprecipitation confirmed that each antibody recognises a distinct form of OPN in both native and denatured states (Figure 1B). MAB53 and MAB194P recognised only their own ~70 kDa and ~80 kDa immunoprecipitate in the blot, and MAB53 also detected an immunoprecipitated band at ~100 kDa which was not apparent in the crude lysate (Figure 1B). MAB222P blotting did not produce a band at ~75 kDa from the MAB222P immunoprecipitate, however two bands at and above 135 kDa were identified (Figure 1B). Each antibody also recognised weaker bands in the lysate, at 35–40 kDa and ≥135 kDa (Figure 1A,B), the former perhaps representative of unmodified polypeptide or cleavage products, and the latter cross-linked oligomers. Notably, the 35–40 kDa polypeptides were not present in any immune-precipitate (Figure 1B).

In summary, all three antibodies bound to a 60 kDa rhOPN, each recognised a sub-fraction of Ishikawa cell OPN, but none of them bound the sub-fraction selected by either of the other two.

### 3.2. Distinct OPN Localisation in Ishikawa Cells Revealed by Three Different Antibodies

**Figure 2:**
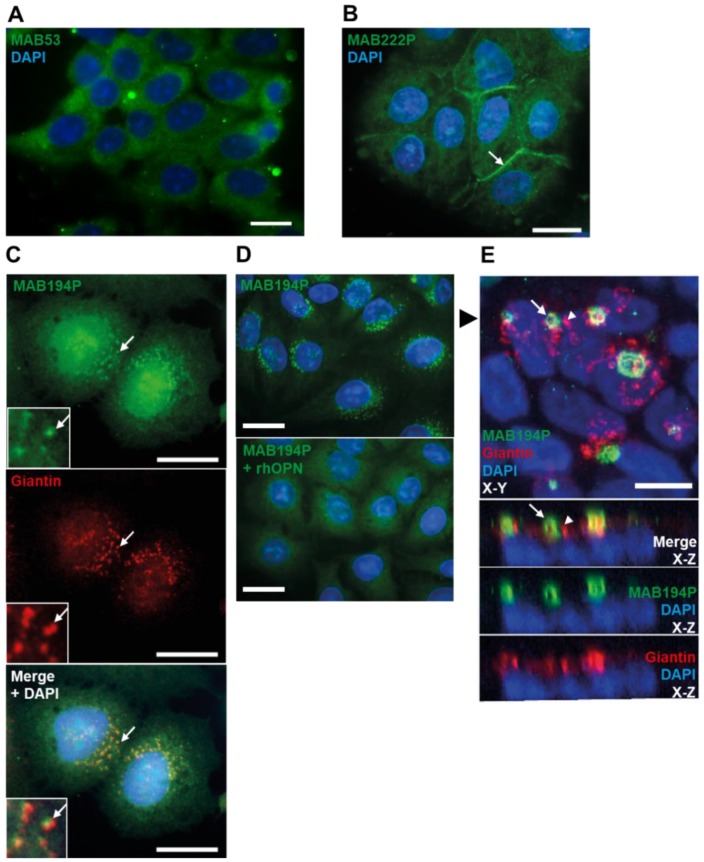
(**A**,**B**) Sub-confluent Ishikawa cells immunostained with MAB53 or MAB222P respectively. 20 µm scale bar. (**C**) Sub-confluent Ishikawa cells co-immunostained with MAB194P (green) and anti-giantin antibody (red). Arrows indicate colocalised puncta. Scale bars are 20 µm. (**D**) Sub-confluent Ishikawa cells immunostained with MAB194P or MAB194P pre-incubated with rhOPN. Scale bars are 20 µm. (**E**) Confluent Ishikawa cell layer co-immunostained with MAB194P (green) and anti-giantin (red). Upper panel is maximum intensity projection of 33 0.24 µm-interval optical sections. Lower panels are Z-plane stacks of optical sections. The black arrowhead in the upper panel indicates the slice of the Z-plane stack. White arrows indicate MAB194P-positive structures and white arrowheads indicate giantin-positive structures. Scale bars are 10 µm.

Immunofluorescence staining of sub-confluent Ishikawa cells with MAB53 produced broad cytoplasmic localisation with exclusion from the nucleus (Figure 2A). In contrast the MAB222P epitope localised to cell borders, which may reflect extracellular membrane localisation (Figure 2B).

Immunofluorescence staining with MAB194P yielded perinuclear, vesicular staining which partially colocalised with the cis-/medial-Golgi marker, giantin (Figure 2C). Using rhOPN to block MAB194P before immunofluorescence led to loss of vesicular staining, demonstrating the specificity of this localisation (Figure 2D). Remarkably, staining confluent Ishikawa cell layers revealed that MAB194P localised to the apical domain in a polarised vesiculotubular pattern largely distinct from the Golgi (Figure 2E). MAB53 and MAB222P localisation was not different in confluent cells (data not shown). These findings corroborate the biochemical data and further suggest that the distinct OPN forms recognised by these antibodies are differentially localised at the subcellular level.

### 3.3. Ishikawa OPN Localisation is not Changed during Interaction with Mouse Blastocysts

**Figure 3:**
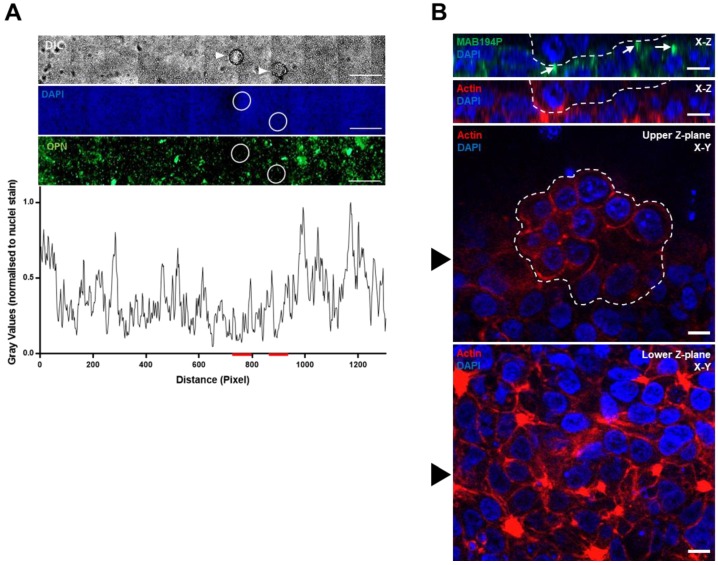
(**A**) Mouse blastocysts were cultured with Ishikawa cell layers for the apposition phase of implantation in vitro (E4.5–5.5). Unattached or weakly attached blastocysts (white arrowheads in top panel) were washed off the Ishkikawa cells before immunostaining with MAB194P. The sites of embryo apposition were identified (white circles). Scale bars are 500 µm. MAB194P intensity normalised to DAPI intensity was measured across the sample and plotted as a line-graph, red lines indicate embryo positions (lower panel). (**B**) Mouse blastocysts stably attached to Ishikawa cells were co-immunostained with MAB194P (green) and phalloidin to label filamentous actin (red). Ten optical sections were captured at 2 µm intervals. Upper panels show Z-plane stacks of optical sections, with white arrows highlighting MAB194P-positive structures. Embryonic cells are delineated by a dashed white line. Lower panels of individual optical sections show subjacent Ishikawa cells and attached embryo. Black arrowhead in lower panels indicates the slice of the Z-plane stacks. Scale bars are 10 µm.

Mouse blastocyst attachment locally increases OPN (MAB53) and integrin αvβ3 immunofluorescence intensity in Ishikawa cells [24]. Here we found that MAB194P staining was not locally changed by mouse blastocysts during the apposition phase, prior to stable attachment and invasion (Figure 3A). Moreover, Ishikawa cells subjacent to, or immediately surrounding attached mouse blastocysts exhibited apical MAB194P staining (Figure 3B), similar to that seen in cells cultured in the absence of embryos (Figure 2E).

### 3.4. Exogenous OPN Inhibits Mouse Blastocyst Invasion of Ishikawa Cells

**Figure 4:**
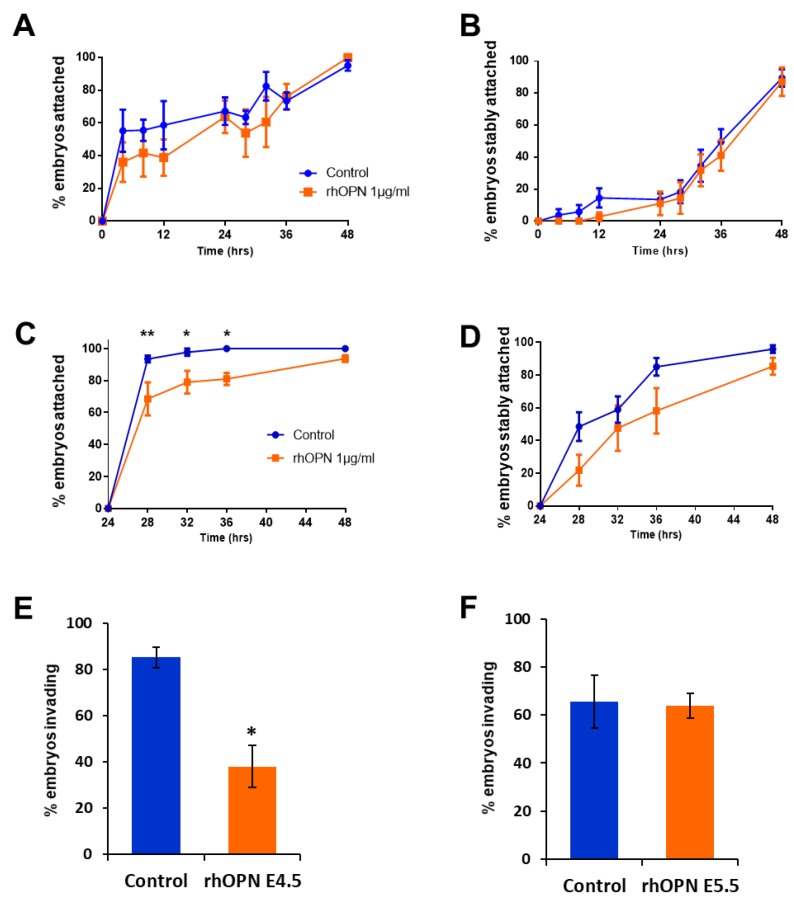
(**A**) RhOPN was added at the onset of embryo–Ishikawa cell co-culture (E4.5), during the apposition phase. Mean percent ±SEM attached embryos from three independent experiments using 12–24 embryos per group. (**B**) Mean percent ±SEM stably attached embryos in the dataset shown in (A). The control group of embryos for (**A**) and (**B***)* was run in parallel with a previously published experiment [30]. (**C**) RhOPN was added at the onset of stable embryo attachment, after 24 h prior co-culture (E5.5). Any weakly attached embryos were dislodged before addition of rhOPN. Mean percent ±SEM attached embryos from four independent experiments using 12 embryos per group; * *p* < 0.05, ** *p* < 0.01 ANOVA. (**D**) Mean percent ± SEM stably attached embryos from (**C**). (**E**) After 48 h (E6.5), co-cultures with rhOPN added during the apposition phase (E4.5) were immunostained with phalloidin and DAPI and imaged to determine embryonic invasion of the Ishikawa cell layer. Mean percent ±SEM invading embryos from three independent experiments from a total of 77 embryos; * *p* < 0.05 independent t-test. (**F**) Co-cultures with rhOPN added just prior to stable attachment (E5.5) were immunostained at E6.5 with phalloidin and DAPI and imaged to assess embryonic invasion. Mean percent ±SEM invading embryos from four independent experiments from a total of 70 embryos.

Endogenous OPN in Ishikawa cells contributes to mouse blastocyst attachment in our in vitro model of implantation [24]. To assess whether exogenous OPN affects intercellular interactions during embryo implantation, 1 µg/mL rhOPN was added to embryo–Ishikawa cell co-cultures during either the apposition (first 24 h) or stable attachment (second 24 h) stage of the experiment. rhOPN did not affect weak attachment during the apposition phase or subsequent stable attachment (Figure 4A,B).

rhOPN added at the onset of stable attachment inhibited initial weak attachment and, although there was a trend towards delayed stable attachment, this did not reach significance (Figure 4C,D). Strikingly, rhOPN treatment during apposition significantly reduced the number of embryos invading into the Ishikawa cell layer, whereas rhOPN treatment during stable attachment did not affect invasion (Figure 4E,F). 

### 3.5. Exogenous OPN Regulates Mouse Blastocyst Gene Expression during Apposition with Ishikawa Cells

**Figure 5:**
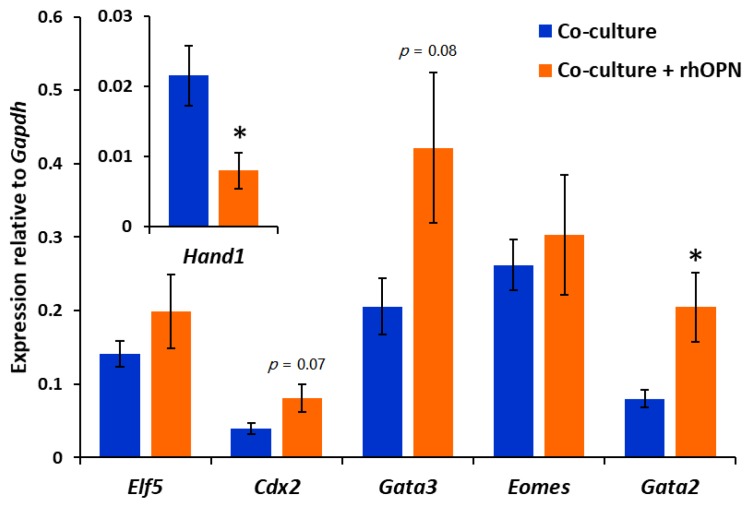
After the apposition phase of co-culture in the presence or absence of rhOPN, embryos were collected and analysed for gene expression by reverse transcription (RT)-qPCR. Mean ±SEM expression level relative to *Gapdh*; five independent experiments consisting of 10 embryos per group. Independent t-test for rhOPN-treated embryos compared to control embryos; * *p* < 0.05, *p* < 0.1 value displayed on graph.

Contact with Ishikawa cell layers during the apposition phase activates mouse blastocyst invasion potential through the regulation of transcription factor expression in the trophectoderm [30]. Blastocysts were collected from co-cultures after apposition in the absence and presence of rhOPN, and expression of a panel of trophectoderm transcription factors was analysed. There was a trend towards upregulation of *Cdx2* and *Gata3* during apposition in the presence of rhOPN, however this did not reach significance. Notably, *Gata2* was significantly upregulated, whereas *Hand1* was downregulated (Figure 5).

## 4. Discussion

Epithelial OPN is one of the biomarkers most consistently associated with endometrial receptivity across species [11]. In ruminants, OPN acts as a bridging ligand in adhesions between uterine luminal epithelium and trophectoderm [22], however, the function of OPN in invasive implantation has not been determined. The present study used monoclonal antibodies to reveal distinct OPN forms in the receptive Ishikawa cell line and identified a vesicular compartment of OPN at the apical domain of polarised epithelial layers of Ishikawa cells. Notably, exogenous OPN added to mouse blastocyst–Ishikawa cell co-cultures inhibited initial attachment interactions, as well as embryonic invasion, in this model of implantation. Furthermore, co-culture with exogenous OPN altered the expression of trophectoderm transcription factors known to control formation of the invasive trophoblast. We propose that OPN acts in a signalling capacity that regulates trophectoderm differentiation during early invasive implantation, although there may be specific effects of endometrial OPN that remain to be determined.

The presence of at least seven OPN forms in the 70–135 kDa range in Ishikawa cells highlights the extensive and differential modification of this ~300-residue polypeptide. Distinct modification in different cell types has previously been suggested [17], however our immunoprecipitation and Western blot data reveal that each of the three antibodies detects distinct OPN forms in both native and denatured states, consistent with non-conformational epitopes. The antibodies predominantly detected forms that were larger than rhOPN, thus endometrial forms are more highly modified than rhOPN. Additionally, distinct localisations for these forms were observed by immunofluorescence, implying that modifications are linked with intracellular and extracellular localisation.

MAB194P antibody data suggested that an ~80 kDa form of OPN partially localised to the cis-/medial-Golgi apparatus of the secretory pathway, perhaps relating to the ER-Golgi intermediate compartment or trans-Golgi network. Golgi localisation of OPN has previously been observed in neurons and kidney tubule cells [33,34,35]. However, the MAB194P-detected OPN form was found in an apical localisation in confluent Ishikawa cells, almost completely separate from cis-/medial-Golgi, which may represent a trans-/post-Golgi compartment in polarised epithelial cells poised or engaged in significant OPN secretion. In mid-secretory phase endometrial tissue, OPN accumulates in a subapical compartment in the luminal epithelium that is known to be highly enriched in secretory vesicles [12,36]. Ishikawa cells are recognised to recapitulate endometrial epithelium in a receptive state [30], thus apical OPN compartments may reflect this. In addition, co-culture with mouse blastocysts did not affect local MAB194P-detected OPN immunoreactivity or localisation, in contrast to previous findings using MAB53 [24]. OPN may therefore alter its subcellular localisation as morphology changes in Ishikawa cells, specifically when organisation into confluent epithelial layers occurs.

MAB222P detected a ~75 kDa form, but only in the denatured state (not immunoprecipitated), while two higher molecular weight forms approximately consistent in size with a covalent dimer were detected in both the native and denatured state. MAB222P immunostaining therefore likely showed the high molecular weight forms and illuminated a cell border localisation which may reflect secreted OPN. However, our previous biochemical survey of the apical surface of Ishikawa cells did not yield OPN as a candidate [37]. Low molecular weight OPN forms are putative cleavage products [38] and were detected here by Western blot but not immunoprecipitation, suggesting native conformations of these forms are not recognised by the antibodies used here. MAB53 has been widely used to analyse OPN and has been shown to bind a mid-peptide epitope lost after specific proteolysis [39]. MAB53 recognised two prominent ~40 kDa bands in Ishikawa cells by Western blot, which may relate to unprocessed forms of OPN as this molecular weight correlates with OPN polypeptide mass. MAB194P and MAB222P also detected a band at ~40 kDa, and MAB222P was raised against the same epitope as MAB53, further suggesting that this band was unprocessed OPN. The MAB194P epitope resides in the N-terminus of the protein, thus smaller forms detected by this antibody could reflect N-terminal OPN cleavage products.

Testing the effect of extracellular OPN in an in vitro embryo implantation model is relevant both to achieving a greater understanding of the role of the endogenous ligand, and in addressing whether its exogenous addition might affect implantation in clinical settings. Mouse blastocysts co-cultured with human endometrial Ishikawa cells is a well characterised implantation model useful for generating hypotheses for testing in more sophisticated primary human cell systems. RhOPN, produced in mouse myeloma cells, was used at a concentration (1 µg/mL) previously shown to activate integrins and stimulate downstream signalling pathways in embryos [25]. OPN is present in foetal bovine serum used to grow Ishikawa cells and although the medium was free from serum during the co-culture phase of the experiments, bovine OPN may be present at the embryo–Ishikawa interface. Although the form of OPN used may not reflect that of human endometrium, we found that exogenous OPN added just prior to the onset of irreversible stable attachment of blastocysts (E5.5) inhibited the initial weak attachment phase, but this effect was not sufficient to delay the attainment of stable attachment. Interestingly, reversible weak attachment seen during the apposition stage from E4.5–5.5 was not affected by exogenous OPN, suggesting reversible and irreversible weak attachment are mediated by different factors. These effects may suggest multiple OPN interactors at the embryo–endometrial interface. Ishikawa cells require endogenous OPN expression for stable embryo attachment [24], however, high concentrations of non-endometrial exogenous OPN may block attachment through altered interactions due to its tissue-specific modifications. OPN receptors integrin αvβ3 and CD44 are expressed in human receptive phase endometrium and in the trophectoderm [12,40,41,42,43]. Our recent evidence suggests that CD44 contributes to both reversible and irreversible weak attachment in a manner independent of its alternative ligand, HA [31]. Together, the data point to a need to investigate endometrial-specific OPN forms, integrin αvβ3 and CD44 as well as other cell surface receptors in attachment interactions at implantation. 

We have previously shown that Ishikawa cells induce mouse trophectoderm differentiation to invasive trophoblast giant cells (TGCs) during the apposition phase, and this is required for embryonic breaching of the epithelium. Downregulation of *Cdx2* and *Gata3* and upregulation of *Hand1* in trophectoderm through Ishikawa cell contact during apposition was proposed to mediate this effect [30]. Herein, the presence of exogenous OPN during apposition inhibited subsequent embryonic invasion, while changes in the expression of trophectoderm transcription factors known to regulate trophoblast differentiation were also observed. *Cdx2*, *Gata3* and *Gata2* are thought to regulate a proliferative trophoblast state [44], and their increased expression in response to exogenous OPN may point to blocked formation of terminally differentiated TGC. Similarly, inhibition of *Hand1* expression by exogenous OPN may represent suppression of TGC differentiation as *Hand1* is an early TGC transcription factor [45]. The potential for OPN to alter trophoblast lineage allocation implies a regulatory role which could be important to balance populations of proliferating and invasive trophoblasts during preimplantation embryo transition to maternally recognised conceptus. External stressors disrupt this balance and induce excessive TGC allocation, which is associated with pregnancy failure [46,47].

Signalling downstream of OPN may include FAK, mitogen-activated protein kinase (MAPK), and mammalian target of rapamycin (mTOR), as these pathways are known to be activated by OPN through integrins in ovine and porcine trophectoderm [48,49,50]. Correspondingly, MAPK has been shown to regulate *Cdx2* and *Gata3* expression [51], and mTOR activation is implicated in trophectoderm differentiation to invasive trophoblast in mouse embryos [52,53]. Moreover, integrin co-receptor IGF1R is thought to function in both embryos and endometrial epithelial cells at implantation through PI3K and FAK, respectively [54,55], and this could be modulated by OPN [56].

## 5. Conclusions

We conclude that secretion of OPN by receptive endometrial epithelium is likely to be highly regulated and that OPN has a significant signalling role at implantation. However, these data do not make a clear case for clinical use of exogenous OPN to promote implantation.

## Figures and Tables

**Table 1:** Details of antibodies used in this study.

Antibody (Clone/Catalogue Number)	Source
OPN (MAB53)	Assay Design
OPN (MAB194P)	Maine Biotechnologies
OPN (MAB222P)	Maine Biotechnologies
Giantin (24586)	Abcam
β-Actin (4967)	Cell Signalling Technologies
Mouse serum IgG (I8765)	Sigma

**Table 2:** Details of PCR primers used in this study.

Gene	Primers (5′–3′)
*Cdx2*	CAAGGACGTGAGCATGTATCC GTAACCACCGTAGTCCGGGTA
*Gata3*	CTCGGCCATTCGTACATGGAA GGATACCTCTGCACCGTAGC
*Eomes*	GCGCATGTTTCCTTTCTTGAG GGTCGGCCAGAACCACTTC
*Elf5*	ACCGATCTGTTCAGCAATGAAG CGCTTGGTCCAGTATTCAGG
*Gata2*	CACCCCGCCGTATTGAATG CCTGCGAGTCGAGATGGTTG
*Hand1*	CTACCAGTTACATCGCCTACTTG ACCACCATCCGTCTTTTTGAG
*Gapdh*	AGGTCGGTGTGAACGGATTTG GGGGTCGTTGATGGCAACA
